# Developing the novel diagnostic model and potential drugs by integrating bioinformatics and machine learning for aldosterone-producing adenomas

**DOI:** 10.3389/fmolb.2023.1308754

**Published:** 2024-01-04

**Authors:** Deshui Yu, Jinxuan Zhang, Xintao Li, Shuwei Xiao, Jizhang Xing, Jianye Li

**Affiliations:** ^1^ Department of Urology, Air Force Medical Center, Beijing, China; ^2^ China Medical University, Shenyang, China

**Keywords:** aldosterone-producing adenomas, primary aldosteronism, artificial neural network, machine learning algorithm, potential targeted drugs

## Abstract

**Background:** Aldosterone-producing adenomas (APA) are a common cause of primary aldosteronism (PA), a clinical syndrome characterized by hypertension and electrolyte disturbances. If untreated, it may lead to serious cardiovascular complications. Therefore, there is an urgent need for potential biomarkers and targeted drugs for the diagnosis and treatment of aldosteronism.

**Methods:** We downloaded two datasets (GSE156931 and GSE60042) from the GEO database and merged them by de-batch effect, then screened the top50 of differential genes using PPI and enriched them, followed by screening the Aldosterone adenoma-related genes (ARGs) in the top50 using three machine learning algorithms. We performed GSEA analysis on the ARGs separately and constructed artificial neural networks based on the ARGs. Finally, the Enrich platform was utilized to identify drugs with potential therapeutic effects on APA by tARGseting the ARGs.

**Results:** We identified 190 differential genes by differential analysis, and then identified the top50 genes by PPI, and the enrichment analysis showed that they were mainly enriched in amino acid metabolic pathways. Then three machine learning algorithms identified five ARGs, namely, SST, RAB3C, PPY, CYP3A4, CDH10, and the ANN constructed on the basis of these five ARGs had better diagnostic effect on APA, in which the AUC of the training set is 1 and the AUC of the validation set is 0.755. And then the Enrich platform identified drugs tARGseting the ARGs with potential therapeutic effects on APA.

**Conclusion:** We identified five ARGs for APA through bioinformatic analysis and constructed Artificial neural network (ANN) based on them with better diagnostic effects, and identified drugs with potential therapeutic effects on APA by tARGseting these ARGs. Our study provides more options for the diagnosis and treatment of APA.

## Introduction

Primary aldosteronism (PA) is caused by adrenocortical lesions characterized by the autonomous secretion of aldosterone. Dysregulation of excess aldosterone causes patients to be at high risk of refractory hypertension, severe hypokalemia or related cardiovascular morbidity and mortality (FUNDER et al., 2016). APA are a common cause of PA (FUNDER et al., 2016; LALLI et al., 2016; WILLIAMS and REINCKE, 2022), a tumor of the adrenal glands that overproduces aldosterone, leading to PA. APA is one of the specific pathologic conditions that lead to primary aldosteronism, but not all primary aldosteronism is caused by APA; other possible causes include adrenocortical hyperplasia (in which the number of cells in the adrenal cortex is increased, leading to an overproduction of aldosterone) or, very rarely, adrenocortical carcinoma (PLOUIN and JEUNEMAITRE, 2004; WILLIAMS and REINCKE, 2018; NANBA and RAINEY, 2021). In recent years, numerous studies have revealed a number of genetic mutations that drive APA development, such as KCNJ5 (CHOI et al., 2011; KITAMOTO and NISHIKAWA, 2022), ATP1A1 (BEUSCHLEIN et al., 2013), ATP2B3 (BEUSCHLEIN et al., 2013), CACNA1D (AZIZAN et al., 2013; SCHOLL et al., 2013), CACNA1H (SCHOLL et al., 2015) and CLCN2 (FERNANDES-ROSA et al., 2018; SCHOLL et al., 2018). These mutations usually lead to abnormalities in cell membrane voltage-gated ion channels, which increase aldosterone synthesis and secretion (OKI and GOMEZ-SANCHEZ, 2020; SPYROGLOU et al., 2021; SCHOLL, 2022). Despite our deeper understanding of the molecular mechanisms of APA, how to accurately link these mutations to clinical manifestations, as well as to the prognosis of the disease, remains an unresolved issue. In addition, diagnostic and therapeutic options for APA remain limited; diagnosis of APA usually involves a series of steps, including clinical evaluation, biochemical testing, imaging, and possibly confirmatory testing (MONTICONE et al., 2015; LAURENT et al., 2018), but current diagnostic methods are not 100% accurate and rely heavily on the experience of the clinician (PIADITIS et al., 2015; STOWASSER, 2015; BEUSCHLEIN et al., 2017). In terms of treatment, if primary aldosteronism is caused by APA, then surgical removal of the tumor is usually the treatment of choice (AMAR et al., 2010). This surgery, usually performed laparoscopically or robotically assisted, can be effective in resolving the disease, lowering blood pressure and improving hypokalemia (CATENA et al., 2010; STOWASSER, 2015). However, not all patients are candidates for surgical treatment. For some patients who are unable to undergo surgical treatment or have poor surgical outcomes, we still need to find more effective treatments. Therefore, the identification of potential biomarkers capable of diagnosing and treating APA is urgent and necessary.

In review, there are few approaches for the diagnosis and treatment of APA, thus a better understanding of the molecular mechanisms of APA is essential to improve the prognosis, early screening and diagnosis of patients who have APA. In the present study, we attempted to construct a novel ANN model diagnosis and evaluation of APA. First, we used three machine learning algorithms to identify hub DEGs for APA and validated the diagnostic effect of these hub DEGs. We then constructed a novel ANN model for APA diagnosis and validated the accuracy of the ANN model in the validation set. In addition, we revealed the biological pathways played specifically by hub DEGs and screened drugs that may tARGset hub DEGs, providing a new perspective for the diagnosis and treatment of APA.

## Methods

### Data acquisition and preprocessing

**FIGURE 1:**
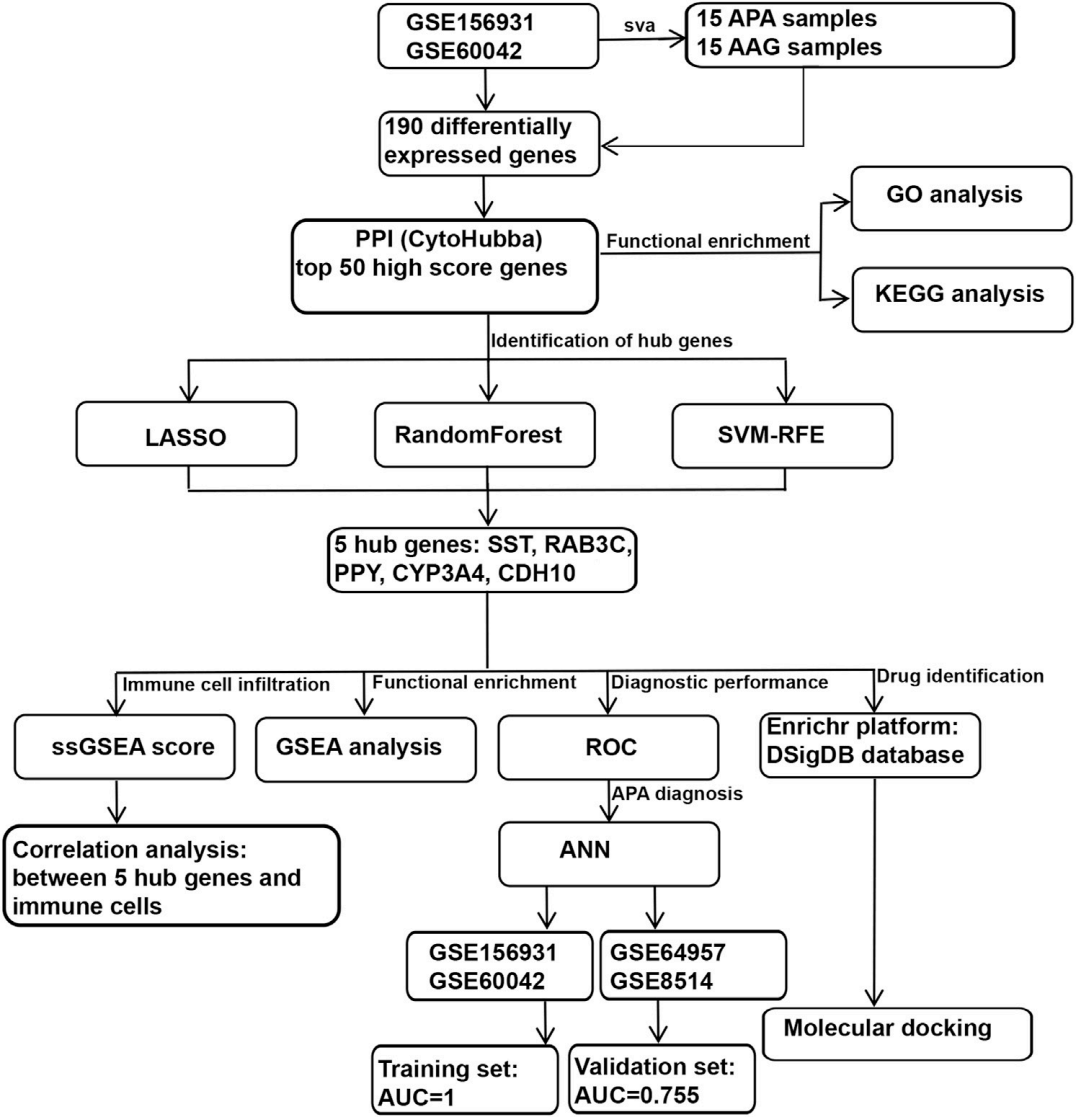
Schematic view of the procedures for data collection and analyses in APA.

The workflow chart of this study is shown in Figure 1. We downloaded 4 APA datasets from the GEO database (https://www.ncbi.nlm.nih.gov/geo/), GSE156931, GSE60042, GSE64957 and GSE8514, respectively. The GSE156931 dataset contains 8 APA and 8 normal adjacent adrenal gland (AAG). The probes were transformed into the corresponding gene symbols by referring to the GPL6883 platform annotation information. The GSE60042 dataset contains 7 APA and 7 AAG. The probes were transformed into the corresponding gene symbols by referring to the GPL14550 platform annotation information. The GSE64957 dataset contains 14 APA and 27 AAG. The probes were transformed into the corresponding gene symbols by referring to the GPL10739 platform annotation information. The GSE8514 dataset contains 10 APA and 5 AAG. The probes were transformed into the corresponding gene symbols by referring to the GPL570 platform annotation information. GSE156931 and GSE60042 were used as the training set. GSE64957 and GSE8514 were used as the validation set for this study. We performed a batch de-effect on GSE156931 and GSE60042. Specifically, we first normalized the gene expression data in the GSE156931 and GSE60042 datasets using the R package “limma”. Subsequently, in order to eliminate the batch effect caused by different platforms, we found that “ComBat” in the R package “sva” could effectively eliminate the batch effect between data generated by different platforms by reviewing previous studies (JOHNSON et al., 2007; TANG et al., 2021). Therefore, we merged the normalized gene expression data of GSE156931 and GSE60042 and used “ComBat” in the R package “sva” to eliminate the batch effect (LEEK et al., 2012). Similarly, GSE64957 and GSE8514 underwent the same batch de-effect.

### Differential analysis of gene expression

Expression profiling data of APA and AAG were compared to identify the differentially expressed genes (DEGs) of 2 clusters using the R package “limma”. The threshold values were |log2FoldChange | >1 and adjusted *p*-value < 0.001, and finally 190 DEGs were obtained.

### PPI and correlation analyses of differentially expressed genes

The STRING database (https://string-db.org/) and Cytoscape software were used to analyse the protein-protein interaction (PPI) among differentially expressed genes (SZKLARCZYK et al., 2019). Differential genes were incorporated into the PPI network, and the differential genes were comprehensively scored using CytoHubba function in Cytoscape, and the top 50 genes with comprehensive scores were finally selected for subsequent analysis.

### Functional enrichment analysis

To clarify which biological processes and functions the top 50 genes are enriched in, to better comprehend the pathogenesis of APA, and we performed Gene Ontology (GO) and Kyoto Encyclopedia of Genes and Genomes (KEGG) analysis of the top 50 genes using the “clusterProfiler” package in R software (YU et al., 2012). A *p*-value of less than 0.05 was set as the cutoff criterion.

### Selection of ARGs

We used 3 machine algorithms to identify ARGs, namely,: randomForest (RF), last absolute shrinkage and selection operator (LASSO) and support vector machine recursive feature elimination (SVM-RFE). First, we used the RF algorithm of “randomForest” package (PAUL et al., 2018), the LASSO algorithm of “glmnet” package (VASQUEZ et al., 2016) and the SVM-RFE algorithm of “e1071” package (NOBLE, 2006) for screening top 50 genes to identify potential candidate genes. Then, we used Venn diagrams to intersect the candidate genes screened by the above 3 algorithms, and finally found 5 intersecting hub candidates.

### Construction and validation of the artificial neural network (ANN) model

We constructed the ANN model using 5 hub genes, which was built using the R package “neuralnet” and consists of 3 parts.

a. Input layer, which includes the gene expression of 5 hub genes;
b. The first hidden layer, which includes the gene expressions of the 5 hub genes and the weights of the 5 hub genes; and the second hidden layer, which includes the weights of all neurons in hidden layer 1.
c. Output layer, which indicates whether the sample belongs to AAG or APA.

To speed up the convergence and improve the accuracy of the neural network, we set the number of neurons in the first hidden layer to 12 and the number of neurons in the second hidden layer to 8, and use ROC to evaluate the prediction performance of the ANN in the training and validation sets.

### ssGSEA

The ssGSEA was performed in R language using the R packages “GSVA” and “GSEABase”, and using the ssGSEA algorithm to evaluate the immunological characteristics among APA patients, respectively. We first obtained 28 immune gene sets from the TISIDB database (http://cis.hku.hk/TISIDB/), and then performed single-sample gene set enrichment analysis (ssGSEA) based on these 28 immune gene sets, and the ssGSEA score of 28 immune gene sets in each sample were calculated.

### Evaluation of the diagnostic value of the selected ARGs in APA

We further investigated whether the selected 5 hub genes are potentially valuable in the diagnosis of APA. The performance of 5 hub genes was evaluated. We performed ROC analysis using the R package “pROC” to obtain AUC, specifically, we obtained 5 hub genes expression data and disease status grouping data from APA sets, performed ROC analysis using the “roc” function of “pROC” package and using the “ci” function of “pROC” to obtain the final AUC results.

### Gene set enrichment analysis

To further identify which biological functions and signaling pathways are associated with 5 hub genes, we clustered APA according to the median value of 5 hub genes expression and performed gene set enrichment analysis (GSEA) on different subgroups, and with *p* < 0.05 being statistically significant.

### Selection and docking of drugs tARGseting 5 hub genes

To screen the drugs tARGseting 5 hub genes, we used the Enrichr platform (https://maayanlab.cloud/Enrichr/) for online analysis and screening. First, we input the gene symbol of 5 hub genes in the primary webpage of Enrichr platform, and then screened the drugs tARGseting 5 hub genes based on the DSigDB database in the “Diseases/Drugs” module, and with *p* < 0.05 being statistically significant. Subsequently, we used molecular docking method (MDM) to investigate the interaction and binding affinity of the screened drug molecules to their hub genes in order to screen for the most potential drugs. Specifically, the molecular structures of potential drugs were acquired from PubChem database (https://pubchem.ncbi.nlm.nih.gov/). Meanwhile, the 3D coordinates of PPY (PDB ID, 4U6S; resolution, 2.10Å), SST (PDB ID, 7T10; resolution, 2.50 Å), CYP3A4 (PDB ID, 5BQG; resolution, 1.44Å), CDH10 (PDB ID, 5VEB; resolution, 2.34Å), and RAB3C (PDB ID, 6Y7G; resolution, 2.30 Å) were retrieved from the PDB (https://www.rcsb.org/). The protein and molecular files were converted to PDBQT format, with the exclusion of water molecules and the inclusion of polar hydrogen atoms. And to enable unrestricted molecular movement, the domain of each protein was encompassed by a centered grid box. AutoDock tools were used to prepare the ligand and protein files. Protein-ligand docking was performed with AutoDock tools, and the resulting interactions between receptor and ligand were visualized with PyMOL (version 2.5).

### Statistical analysis

Statistical analysis and visualization were conducted using R software for this study. The analysis of variance (ANOVA) method was employed to statistically analyse multi-group data, while the wilcoxon rank sum test was used to compare two groups. For all statistical analyses, a significance level of *p* < 0.05 was considered statistically significant. In addition, we have provided the scripts for the main analyses required for this study in the Supplementary Material S1 for consultation.

## Results

### Identification of differentially expressed genes in APA

**FIGURE 2:**
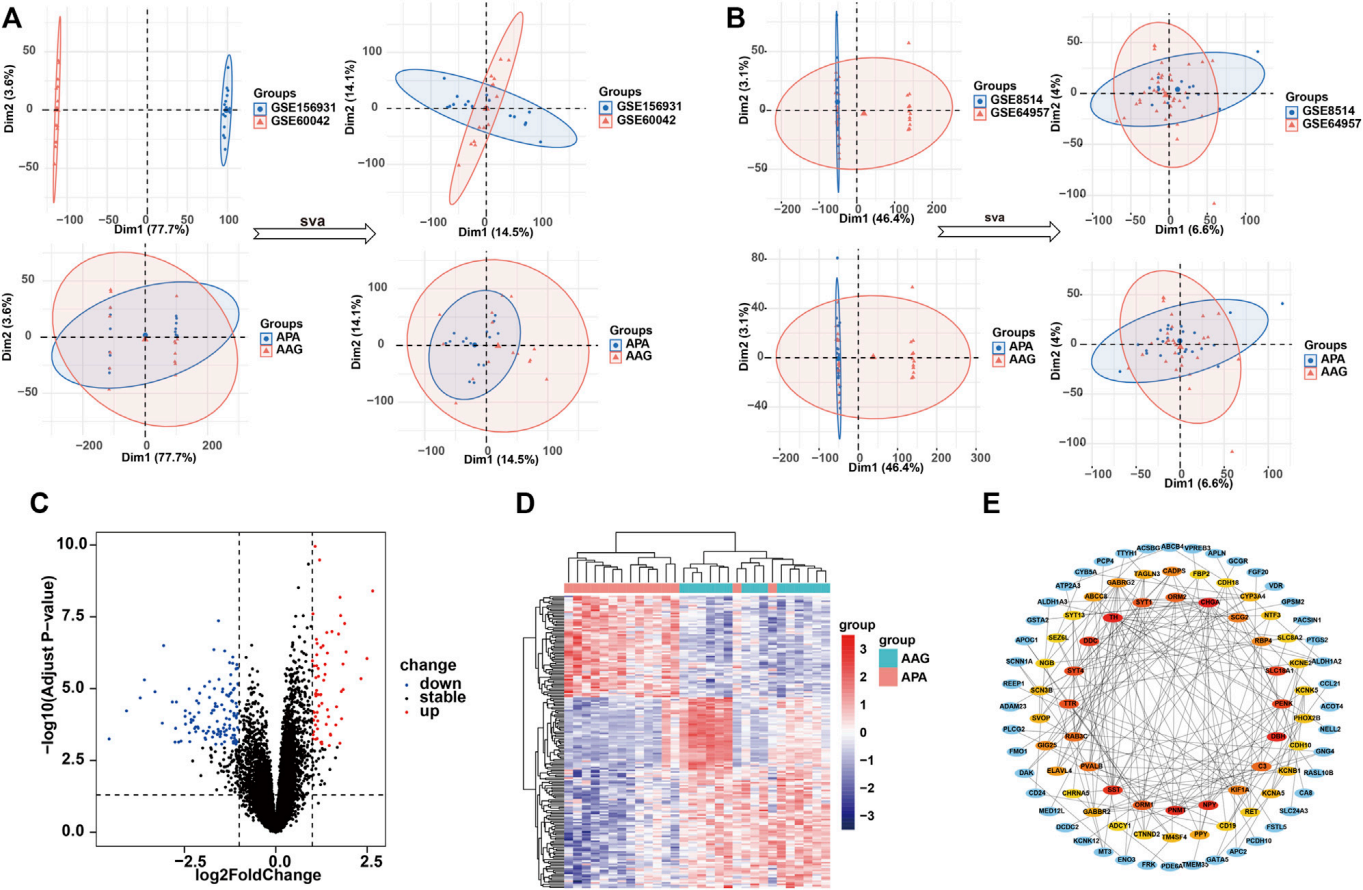
Identification of the DEGs in APA. **(A)** Data characteristics of the training set before and after the debatch effect. **(B)** Data characteristics of the validation set before and after the debatch effect. **(C)** Volcano shows expression of DEGs between the APA and AAG cohort. **(D)** Heatmap shows the overall landscape of DEGs between APA and AAG. **(E)** The protein-protein interaction network shows the interactions between DEGs, with the inner circle showing the top 50 genes with the higher scores.

To systematically identify ARGs that enable the diagnosis and treatment of APA, we conducted a set of analyses. The study design was illustrated in Figure 1. We downloaded the RNA-seq datasets from 15 APA patients and 15 AAG retrieved by GEO datasets (GSE156931 and GSE60042) and performed a de-batch effect on the two datasets to ensure data consistency. The de-batch effect results showed that the data of the dataset grouping and the disease status grouping were consistent (Figure 2A). Additionally, we also performed the de-batch effect on an independent validation set for this study consisting of two datasets, GSE8514 and GSE64957, and the results showed that the treated samples were uniformly dispersed (Figure 2B). Then, we performed differential gene expression analysis, and the results identified a total of 190 differentially expressed genes, of which 66 were upregulated and 124 were downregulated (Figure 2C). The overall landscape of 190 DEGs between APA and AAG is shown in Figure 2D. Next, the protein-protein interaction of these 190 differential genes was constructed using STRING database (https://string-db.org/), and then the CytoHubba plug-in in Cytoscape was utilized for overall evaluation of these 190 differential genes. Finally, the top 50 genes with the highest scores were selected (Figure 2E).

**FIGURE 3:**
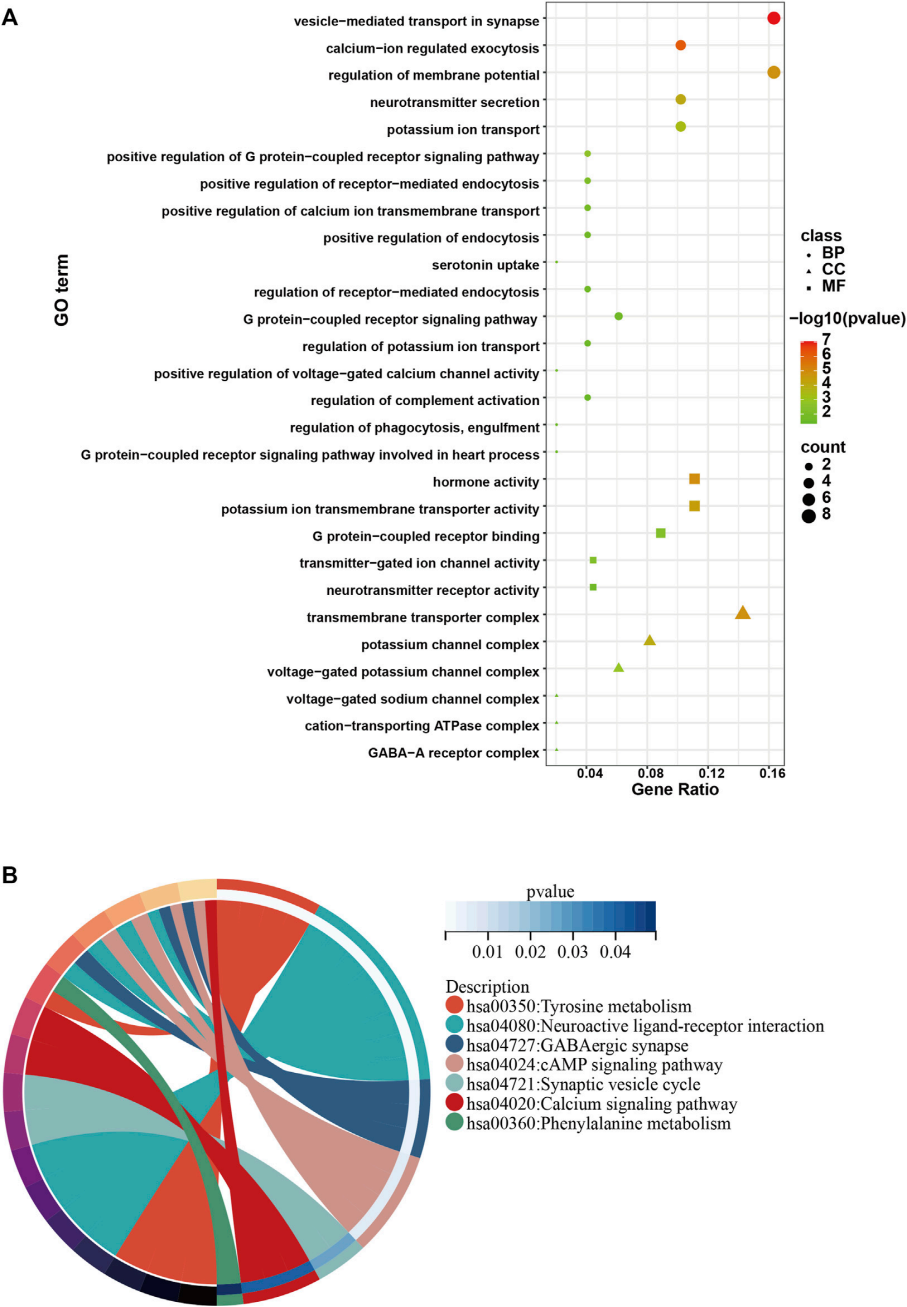
GO and KEGG analysis of the top 50 genes with the higher scores. **(A)** GO enrichment results in the top 50 genes. **(B)** KEGG enrichment results in the top 50 genes.

To investigate the role of the top 50 genes, we performed Gene Ontology (GO) and Kyoto Encyclopedia of Genes and Genomes (KEGG) analysis. Consequently, GO enrichment analysis revealed multiple biological processes, including “voltage−gated potassium channel complex”, “G protein−coupled receptor binding”, “potassium ion transmembrane transporter activity”, “G protein−coupled receptor signaling pathway involved in heart process”, “regulation of phagocytosis, engulfment”, “regulation of complement activation”, “positive regulation of voltage−gated calcium channel activity”, “regulation of potassium ion transport”, “G protein−coupled receptor signaling pathway”, “regulation of receptor−mediated endocytosis”, “positive regulation of endocytosis”, “positive regulation of receptor−mediated endocytosis”, “positive regulation of G protein−coupled receptor signaling pathway” and “potassium ion transport” (Figure 3A). The KEGG enrichment analysis including “Tyrosine metabolism”, “Neuroactive ligand-receptor interaction”, “GABAergic synapse”, “cAMP signaling pathway”, “Synaptic vesicle cycle”, “Calcium signaling pathway” and “Phenylalanine metabolism” (Figure 3B).

### Identification of hub genes using 3 machine learning algorithms

**FIGURE 4:**
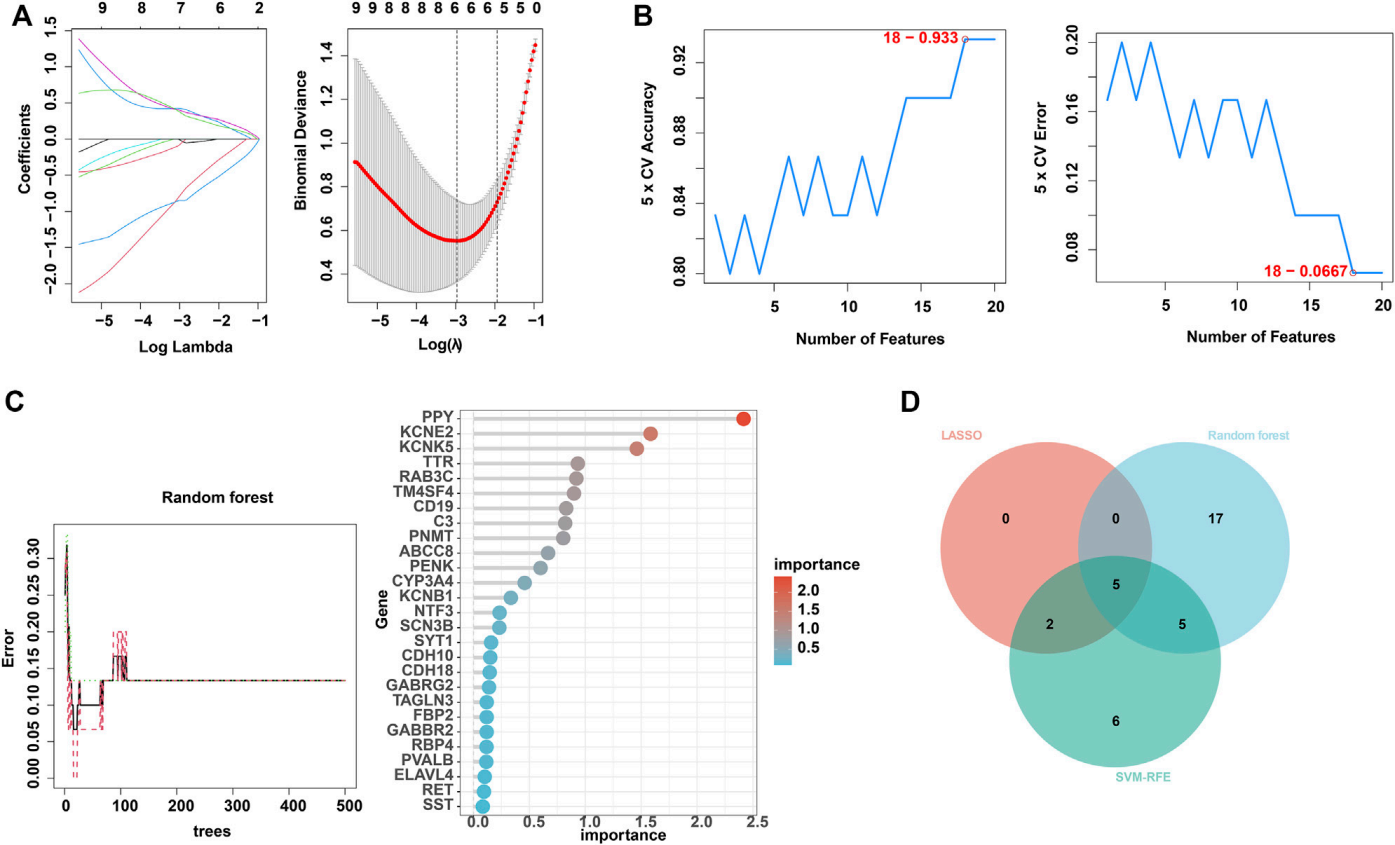
Identification of hub genes using 3 machine learning algorithms. **(A)** LASSO coefficient profiles of the indicated differentially expressed immune-related genes (left panel). After cross-validation for tuning parameter selection, 7 candidate ARGs were identified (right panel). **(B)** SVM–RFE algorithm identified 18 candidate genes with an accuracy of 0.933 (left panel) and an error of 0.0667 (right panel). **(C)** RandomForest algorithm identified 27 candidate genes. RandomForest error rate *versus* the number of classification trees (left panel) and gene importance scores (right panel). **(D)** Venn plot shows the overlapped candidate genes.

Next, to find out the key differentially expressed genes in APA, we applied 3 machine learning algorithms, including least absolute shrinkage and selection operator (LASSO), support vector machine recursive feature elimination (SVM-RFE), and random forest, as these machine learning approaches have been widely employed to analyse biological data and accurately identify hub genes in gene expression profiles (CHEN et al., 2016). Firstly, we utilized the LASSO algorithm to identify the variation in regression coefficients of 50 differentially expressed genes and select the optimal and minimal criteria of the penalization parameter (λ) using 10-fold cross-validation (Figure 4A), and 7 candidate genes were screened (SHAO et al., 2021). Besides, We also established the SVM-RFE model to screen out the genes with the minimum cross-validation error (Figure 4B), and the SVM-RFE algorithm screened 18 candidate genes with an accuracy of 0.933 and an error of 0.0667 (NOBLE, 2006). Meanwhile, we also used the obtained 50 differentially expressed genes to incorporate into the random forest model, the cross-validation error was minimized to 10 trees (Figure 4C). Subsequently, 27 candidate genes with important points than zero were identified by random forest (PAUL et al., 2018). In summary, LASSO algorithm identified 7 candidates, while the SVM-RFE algorithm identified 18 candidates, and the randomForest algorithm identified 27 candidates (Table 1). By intersected all the candidates, we found SST, RAB3C, PPY, CYP3A4 and CDH10 could be identified by all the indicated machine learning approaches and thus defined as hub genes (Figure 4D).

**TABLE 1:** Scanning of candidate machines by 3 machine learning algorithms.

Methods	Genes
Lasso	*TH, SST, RAB3C, PPY, CYP3A4, CTNND2, CDH10*
RandomForest	*PPY, KCNE2, KCNK5, TTR, RAB3C, TM4SF4, CD19, C3, PNMT, ABCC8, PENK, CYP3A4, KCNB1, NTF3, SCN3B, SYT1, CDH10, CDH18, GABRG2, TAGLN3, GABBR2, FBP2, RBP4, PVALB, ELAVL4, RET, SST*
SVM-REF	*CYP3A4, PPY, RAB3C, CTNND2, ORM2, CDH10, DDC, GABRG2, SST, GABBR2, ORM1, KIF1A, TM4SF4, C3, CD19, ADCY1, TH, PHOX2B*

### Diagnostic efficacy of hub genes in predicting APA

**FIGURE 5:**
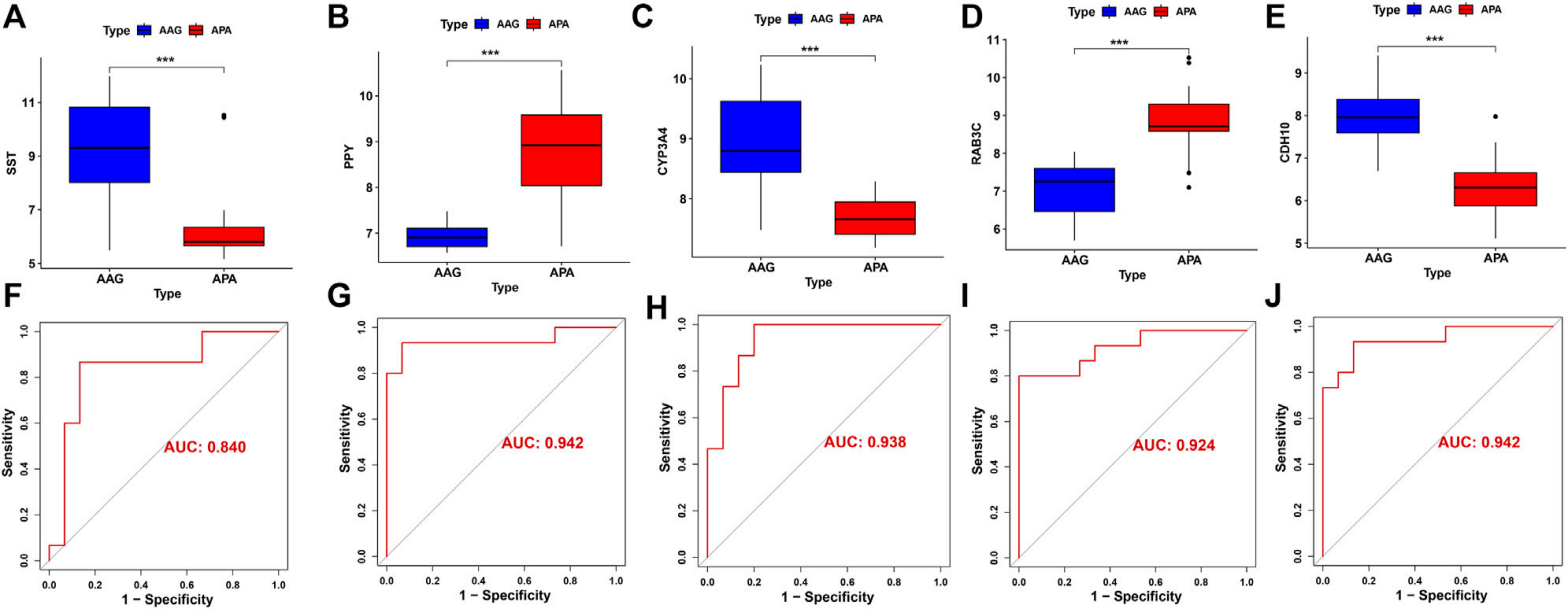
The performance of the signature genes. **(A-E)** The expression of signature genes between the APA and AAG cohort. **(F-J)** ROC showed the diagnostic performance of the signature genes.

**FIGURE 6:**
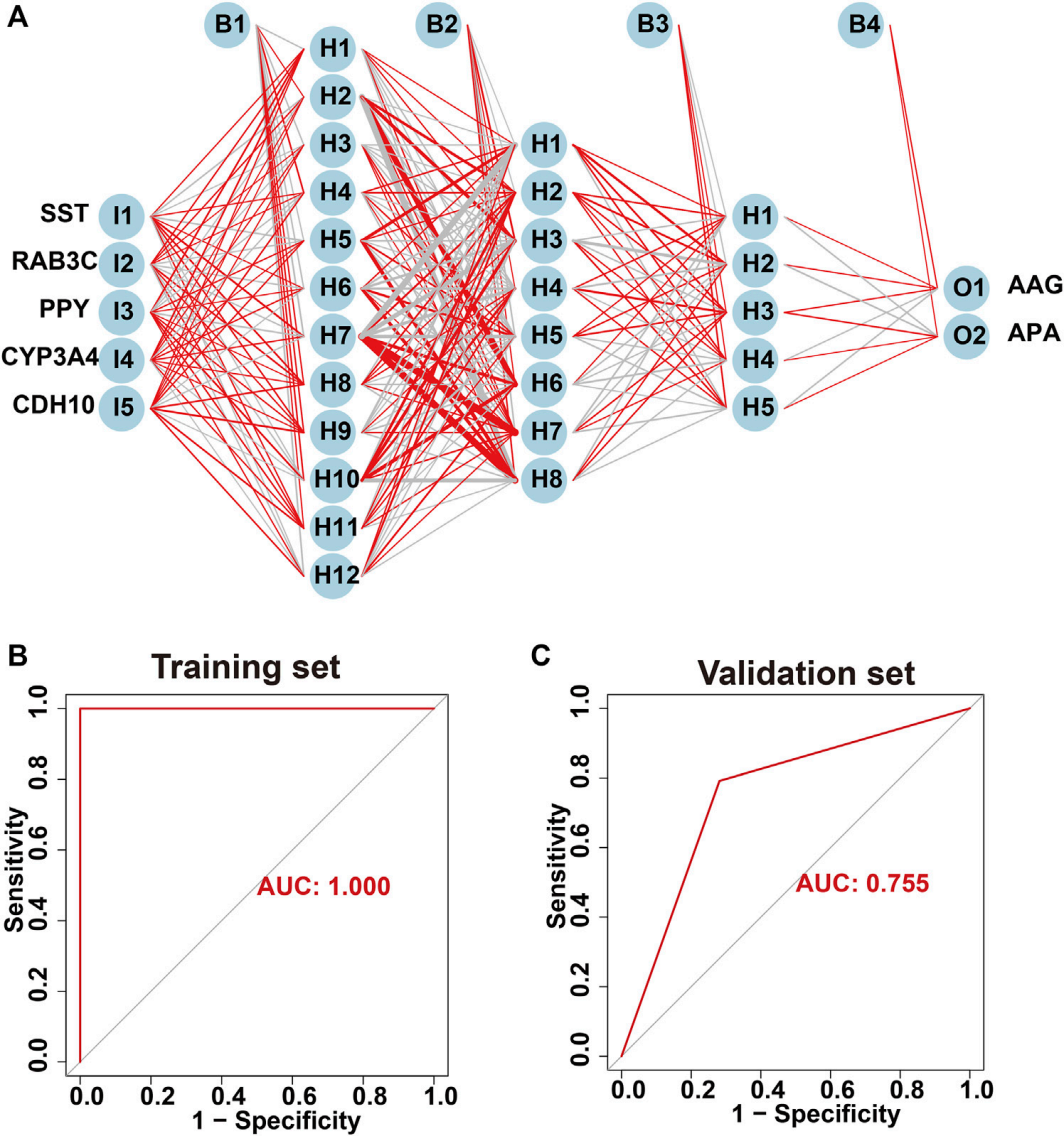
Construction of artificial neural network (ANN) based on hub genes. **(A)** Construction of ANN based on SST, RAB3C, PPY, CYP3A4 and CDH10. **(B)** The AUC of the training set with a value of 1. **(C)** The AUC of the validation set with a value of 1.

The screened hub genes were significantly differentially expressed in APA than those in AAG, suggesting that these genes may play a potential role in contributing APA (Figures 5A–E). Furthermore, the area under curve (AUC) of the receiver operating characteristic curve (ROC) of these hub genes was 0.840 of UPP1, 0.942 of S100A9, 0.938 of KIF1B, 0.924 of S100A12, 0.942 of SLC26A8 respectively (Figures 5F–J). These phenomena indicated that the screened hub genes had remarkable diagnostic efficiency in forecasting APA. Besides, we also constructed ANN based on ARGs to diagnose the onset of APA. ANN is one of the main types of artificial intelligence that has been used in many specialized areas of clinical medicine (CHEN et al., 2018). Specifically, we incorporated hub genes into the artificial neural network and constructed an ANN model to predict whether the samples belonged to AAG or APA, which consisted of three parts: input layer, hidden layer and output layer (Figure 6A). Then we compare the ANN model prediction results with the actual grouping information of the samples. We use two datasets, GSE156931 and GSE60042, as the training set of the ANN and two datasets, GSE64957 and GSE8514, as the validation set of the ANN, respectively. The ANN prediction results and their accuracy for the training and validation sets are shown in Table 2, where the prediction accuracy for the training set is 100% and the prediction accuracy for the validation set is 75%. Finally, we evaluate the prediction capability of the ANN model on the training and validation sets using the ROC curves, where the AUC value for the training set is 1 (Figure 6B) and the AUC value for the validation set is 0.755 (Figure 6C). It was shown that an area under the ROC curve greater than 0.5 proves that the diagnostic model has some diagnostic value (ROBIN et al., 2011). Generally, the ANN model is convincing and has the potential to be used as an independent diagnostic predictor for APA.

**TABLE 2:** ANN diagnosis effect for the training and validation sets.

		Training set		Validation set	
		AAG	APA	AAG	APA
Prediction	AAG	15	0	23	5
	APA	0	15	9	19
	Accuracy	100%		75%	
	AUC	1		0.755	

### GSEA analysis

**FIGURE 7:**
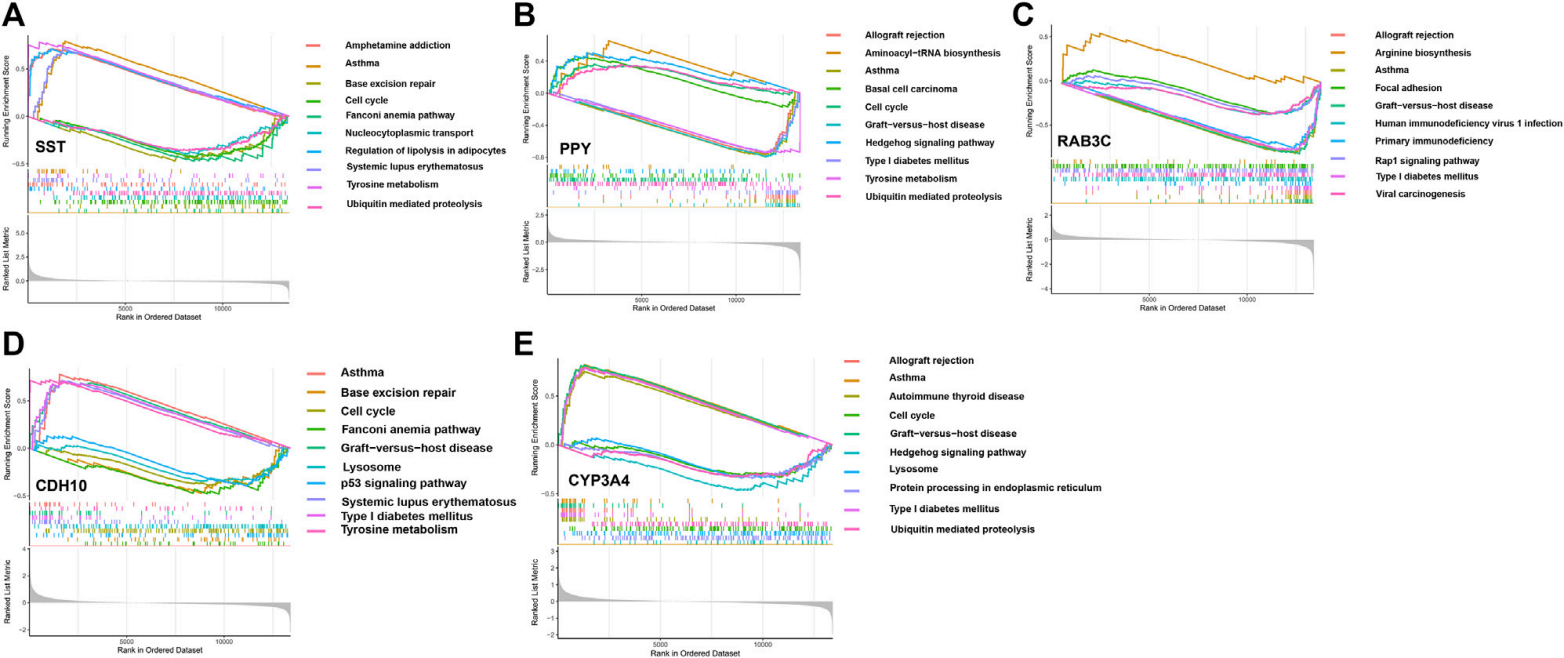
The GSEA of the signature genes in APA. **(A)** The GSEA of BAB3C in APA. **(B)** The GSEA of CDH10 in APA. **(C)** The GSEA of SST in APA. **(D)** The GSEA of CYP3A4 in APA. **(E)** The GSEA of PPY in APA.

We assessed signaling pathways associated with signature genes *via* GSEA analysis. The top 10 signaling pathways were displayed in Figure 7. The results showed that CDH10 was significantly correlated with Asthma, Tyrosine metabolism, Graft-versus-host disease, Type I diabetes mellitus, Systemic lupus erythematosus, Lysosome, Cell cycle, p53 signaling pathway, Base excision repair, Fanconi anemia pathway. The expression of CYP3A4 significantly correlated with Asthma, Graft-versus-host disease, Allograft rejection, Type I diabetes mellitus, Autoimmune thyroid disease, Ubiquitin mediated proteolysis, Cell cycle, Lysosome, Protein processing in endoplasmic reticulum, Hedgehog signaling pathway. The expression of PPY significantly correlated with Aminoacyl-tRNA biosynthesis, Hedgehog signaling pathway, Basal cell carcinoma, Cell cycle, Ubiquitin mediated proteolysis, Tyrosine metabolism, Type I diabetes mellitus, Allograft rejection, Asthma, Graft-versus-host disease. The expression of RAB3C significantly correlated with Arginine biosynthesis, Focal adhesion, Rap1 signaling pathway, Viral carcinogenesis, Human immunodeficiency virus 1 infection, Primary immunodeficiency, Type I diabetes mellitus, Allograft rejection, Asthma, Graft-versus-host disease. The expression of SST significantly correlated with Asthma, Tyrosine metabolism, Systemic, lupus erythematosus, Amphetamine addiction, Regulation of lipolysis in adipocytes, Ubiquitin mediated proteolysis, Nucleocytoplasmic transport, Cell cycle, Base excision repair, Fanconi anemia pathway. Taken together, these genes all positively correlated Cell cycle, Protein processing and proteolysis, Amino acid metabolism signaling pathway as well as tumor-immune signaling pathway.

### Immune cell infiltration

**FIGURE 8:**
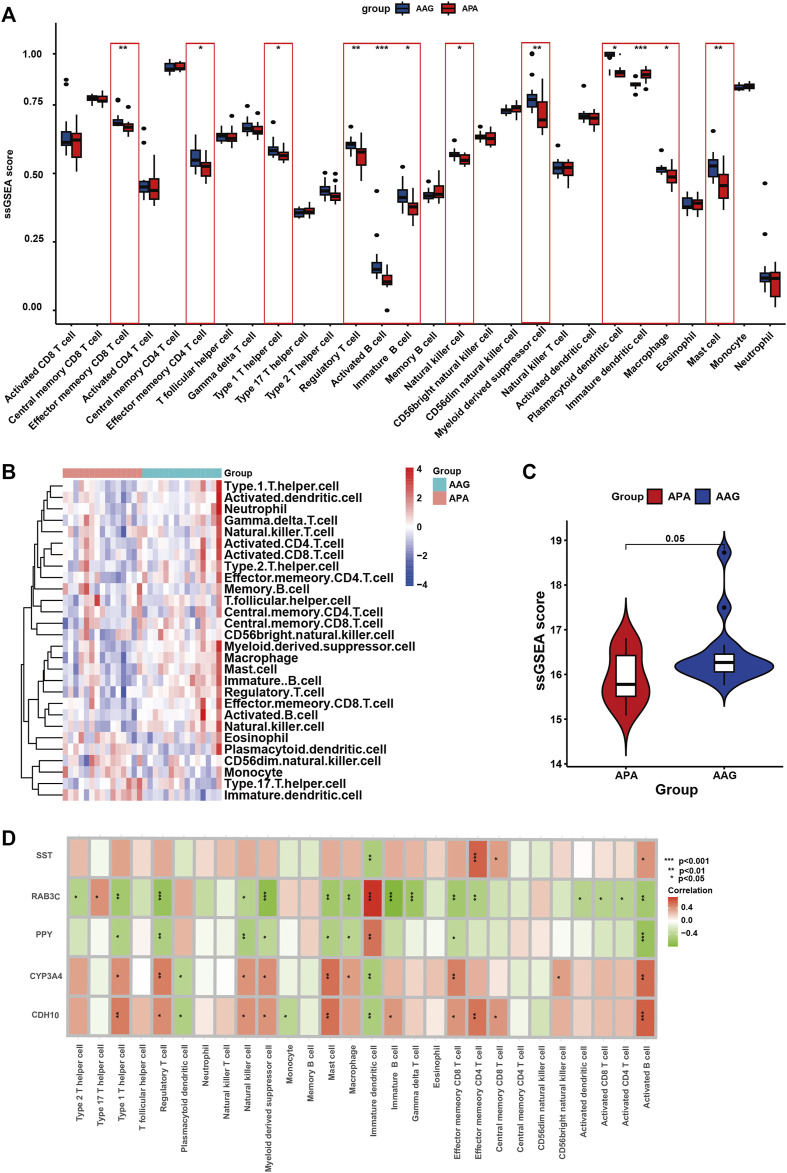
The immune cell infiltration association with signature genes. **(A)** The box plot shows the immune cell infiltration between the APA and AAG groups. **(B)** Heatmap shows the overall landscape of APA and AAG groups’ ssGSEA score. **(C)** The violin plot shows the ssGSEA score of immune cells of the APA and AAG groups. **(D)** The association between signature genes and significantly different immune cell infiltration. (*p < 0.05; **p < 0.01; ***p < 0.001).

Immunological features were evaluated according to immune cell infiltration. Compared with AAG, APA have lower Effector memeory CD8 T cell, Effector memeory CD4 T cell, Type 1 T helper cell, Regulatory T cell, Activated B cell, Immature B cell, Natural killer cell, Myeloid derived suppressor cell, Plasmacytoid dendritic cell, Macrophage, Mast cell (Figure 8A). The overall landscape of immune cell infiltration is shown in Figure 8B, and the results showed significant differences in immune cell infiltration between the APA and AAG groups. Meanwhile, we compared the ssGSEA scores between APA and AAG groups, and the results showed that APA had significantly lower immune cell infiltration than AAG (Figure 8C). All hub genes were correlated with the infiltration of Immature dendritic cell and Activated B cell. CYP3A4 and CDH10 were positively correlated with Type 1 T helper cell, Regulatory T cell, Natural killer cell, Myeloid derived suppressor cell, Mast cell, Effector memeory CD8 T cell and Activated B cell, and negatively correlated with Plasmacytoid dendritic cell, Immature dendritic cell. While RAB3C, PPY were positively correlated with Immature dendritic cell, and negatively correlated with Type 1 T helper cell, Regulatory T cell, Natural killer cell, Myeloid derived suppressor cell, Mast cell, Effector memeory CD8 T cell, Macrophage and Activated B cell. SST was only positively correlated with Effector memeory CD4 T cell, Central memory CD8 T cell, Activated B cell, and negatively correlated with Immature dendritic cell (Figure 8D).

### Identification and docking of potential drugs targeting hub genes

**FIGURE 9:**
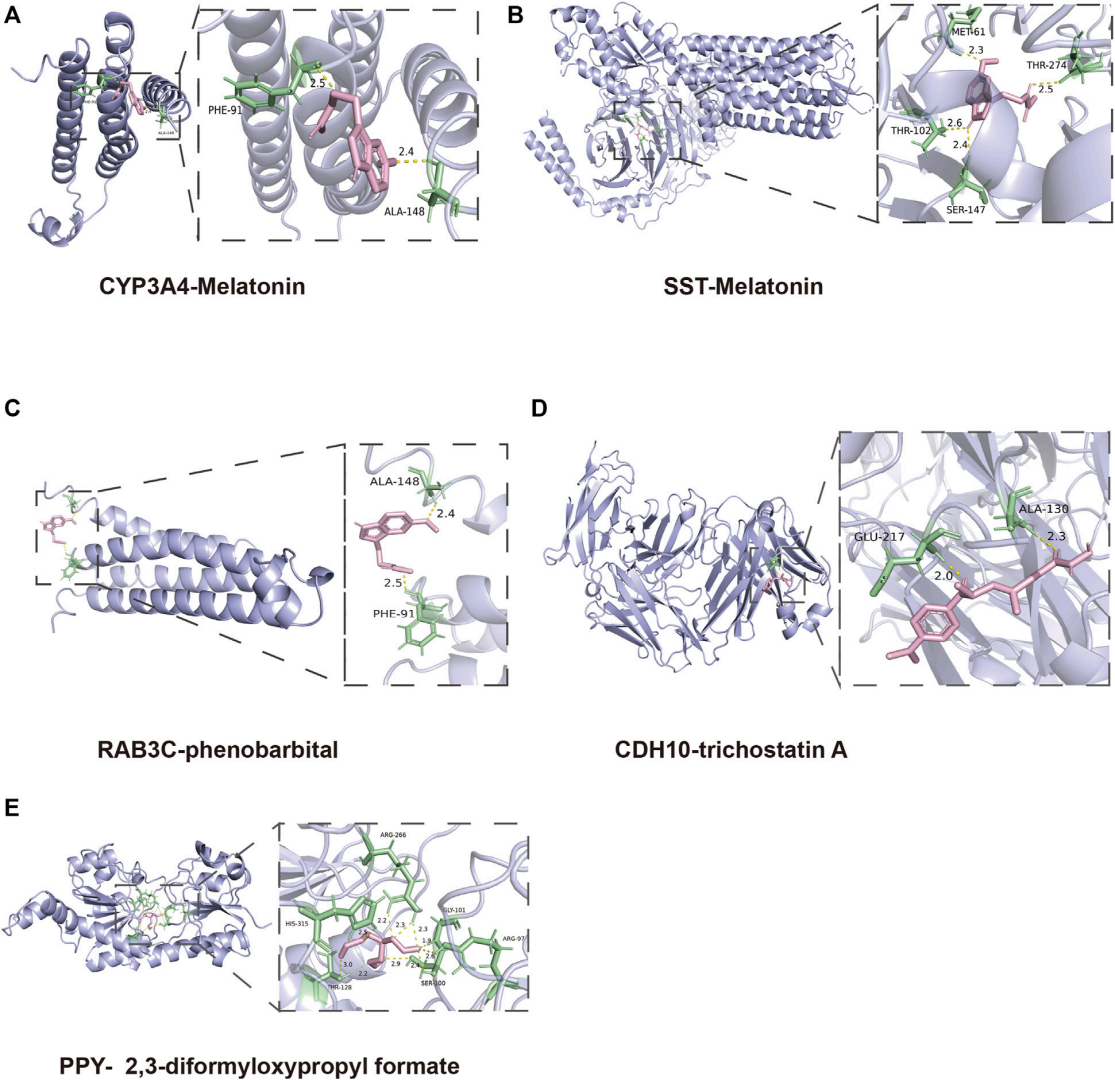
3D structures of the interacted interface between hub genes and their potential binding drugs. **(A, B)** The structure of the complex formed by the docking of Melatonin with SST and CYP3A4. **(C)** The structure of the complex formed by the docking of phenobarbital with RAB3C. **(D)** The structure of the complex formed by the docking of trichostatin A with CDH10. **(E)** The structure of the complex formed by the docking of 2,3-diformyloxypropyl formate with PPY.

To find out the drugs targeting hub genes, we used the Enrichr platform (https://maayanlab.cloud/Enrichr/) for online analysis and screening. We identified four drugs targeting hub genes based on the DSigDB database with a *p*-value < 0.05. Next, we used the molecular docking method (MDM) to investigate the binding affinity of the drugs with their targeting hub genes, and their binding energy is shown in Table 3. The results showed that Melatonin was able to target SST and CYP3A4, in the order of CYP3A4-Melatonin (−6.0 kcal/mol) and SST-Melatonin (-5.7 kcal/mol) (Figures 9A, B). In addition, the absolute values of the binding energies of the two complexes, RAB3C-phenobarbital (−6.3 kcal/mol) and CDH10-trichostatin A (−6.8 kcal/mol), were relatively high (Figures 9C, D), while PPY- 2,3-diformyloxypropyl formate (−5.6 kcal/mol) with relatively low absolute values of binding energy (Figure 9E). Overall, the docking results suggest that these potential drugs may regulate the pathologic development of APA by interacting with hub genes.

**TABLE 3:** Potential drugs tARGseting hub genes.

Term	*p*-value	Combined score	Genes	Binding energy (kcal/mol)
2,3-diformyloxypropyl formate	0.006981041	917.8914803	PPY	−5.6
trichostatin A	0.04316521	21.60762902	CDH10	−6.8
phenobarbital	0.028399813	39.74917525	RAB3C	−6.3
Melatonin	8.27E-04	515.1515385	SST; CYP3A4	−5.7/-6.0

## Discussion

APA is one of the common clinical subtypes of PA, a tumor that autonomously secretes aldosterone mainly from the zona glomerulosa, with hypertension and hypokalemia as the main clinical manifestations may lead to serious cardiovascular complications if untreated (PLOUIN et al., 2004; ZENNARO et al., 2020).

Current clinical diagnostic methods for APA include clinical evaluation, biochemical testing, imaging and possible confirmatory testing. However, there are some drawbacks to these diagnostic methods; for example, in imaging, CT and MRI can show the structure of the adrenal gland and possible tumors, but they cannot distinguish between APA and nonfunctional adrenal tumors, or accurately differentiate between APA and adrenocortical hyperplasia. In addition, these imaging techniques may not be able to detect microscopic adenomas smaller than 1 cm. In addition, confirmatory tests also typically require special conditions (e.g., specific salt intake) and complex sample processing, which may also lead to false-positive or false-negative results in some specific cases (SONOYAMA et al., 2011). In addition, the preferred clinical treatment for APA is surgical resection, but there is still a need to find more effective treatments for some patients who are unable to undergo surgery or who have poor surgical outcomes (BRAVO et al., 1988; LO et al., 1996).

In other words, there is an urgent need for potential biomarkers and tARGseted drugs for the diagnosis and treatment of APA.

In the present study, we first screened 190 DEGs from 15 APA patients and 15 healthy controls based on the GEO databases GSE156931 and GSE60042. Subsequently, in order to find tightly interacting genes among these 190 DEGs, we included them in the PPI interaction interactions network and took the top 50 genes with the highest composite scores to be included in the subsequent analysis. To explore the signaling pathways and biological functions that may contribute to the development of APA, we performed GO and KEGG enrichment analyses on the top 50 genes with the highest scores. Our enrichment analysis indicated that the pathogenesis of APA may be related to amino acid metabolism, Calcium signaling pathway, G protein-coupled receptor signaling pathway, and GABAergic synapse.

To identify ARGs in APA, we used three machine learning algorithms, including LASSO, SVM-RFE, and Randomforest. We found that SST, RAB3C, PPY, CYP3A4, CDH10 are five ARGs in APA, and they were identified by all the machine learning methods, suggesting that they have a potential role in the development of aldosterone tumor and development of APA. Certain of these genes have been reported in previous studies, for example, RAB3C is a peripheral membrane protein that is involved in membrane trafficking (vesicle formation) and cell movement (CHANG et al., 2023), and RAB3C overexpression promotes tumor metastasis and is associated with poor prognosis in colorectal cancer, through modulating the ability of cancer cells to release IL-6 through exocytosis and activate the JAK2-STAT3 signaling pathway (CHANG et al., 2017). Also, CDH10 is involved in sporadic pancreatic carcinogenesis, and might have a role in rare cases of familial pancreatic cancer (JINAWATH et al., 2017).

These 5 ARGs are probably mechanistically involved in the onset and development of APA, then they can also be potential diagnostic tARGsets for APA. Of course, the diagnostic performance of these 5 ARGs for APA still needs to be verified by artificial neural network modeling (MANDAIR et al., 2023). At present, the clinical approach for diagnosing APA still has some drawbacks. Therefore, we incorporated these 5 ARGs into the artificial neural network and constructed an ANN model to predict whether the samples in this study belonged to the healthy control or APA, in which the prediction accuracy for the training set is 100% and the prediction accuracy for the validation set is 75%. At the same time, we evaluate the prediction capability of the ANN model on the training and validation sets using the ROC curves, where the AUC value for the training set is 1 and the AUC value for the validation set is 0.755. That is, the ANN has the potential to be used as an independent diagnostic predictor for APA.

Meanwhile, we also performed a comprehensive assessment of the tumor microenvironment (TME) of APAs in this study, and we demonstrated the different cellular components of the TME between APAs and AAGs. Our study showed a lower density of CD45 lymphocyte infiltration in APAs. Our study showed a lower density of tumor-infiltrating CD45 lymphocytes in APAs. This change in the TME in APAs may be due to competition for nutrients in the TME and the formation of an acidic microenvironment by glycolytic intermediates that inhibits immune activation (CHANG et al., 2015; LEONE and POWELL, 2020). Another potential mechanism is the activation of PPAR signaling to promote evasion of immune surveillance (KORPAL et al., 2017). For example, PPARα has anti-inflammatory activity, and PPARα agonists mediate a variety of immune responses that can reverse acute and chronic liver inflammation (PAWLAK et al., 2015). In addition, we observed lower infiltration of effector memory CD8 T cells in APA, and it has been previously shown that effector T cell viability is reduced in glucose-limited *in vitro* medium (ZHAO et al., 2016), suggesting that metabolic reprogramming of glycolysis in APA may create a hypoglycemic environment and limit glucose uptake by immune cells, thereby hindering their function.

Certainly, the present study has some limitations. We constructed an ANN diagnostic model based on the transcriptomes of only 30 samples from the GEO database, and although the reliability of the model was verified in the data set independent of the present study, the present study is still at a relatively early stage, and many clinical trials are needed to validate the diagnostic model if it is truly applied to assist clinical diagnosis. Of course, the present study also provides promising targets for the diagnosis and treatment of APA at the transcriptome level, and we aim to continue our work on the diagnosis and treatment of APA through the integration of multi-omics (transcriptome, proteome, metabolome, etc.) in our future work.

In review, there are few methods for diagnosing and treating APA, thus there is an urgent need for potential biomarkers and tARGseted drugs for the diagnosis and treatment of APA. Specifically, we first used three machine learning to identify ARGs in APA and validated the diagnostic effect of these ARGs, meanwhile we constructed a novel ANN model for APA diagnosis and validated the accuracy of this ANN model in validation set. We then explored the immune infiltration between the two groups of APA patients and healthy controls and assessed the correlation between these five ARGs and differential immune cells. Finally, we identified drugs that may tARGset these 5 ARGs through the Enrich platform, providing new perspectives for the diagnosis and treatment of APA.

## Data Availability

The original contributions presented in the study are included in the article/Supplementary Material, further inquiries can be directed to the corresponding author.

## Abbreviations

APA, Aldosterone-producing adenomas; AAG, Adjacent adrenal gland; ARGs, Aldosterone adenoma-related genes; PA, Primary aldosteronism; GEO, Gene expression omnibus; ANN, Artificial neural network; DEGs, Differentially expressed genes; AAG, adjacent adrenal gland; PPI, Protein-protein interaction; GO, Gene Ontology; KEGG, Kyoto Encyclopedia of Genes and Genomes; LASSO, Last absolute shrinkage and selection operator; SVM-RFE, Support vector machine recursive feature elimination; ssGSEA, Single-sample gene set enrichment analysis; ROC, Receiver operating characteristic; AUC, Area under the ROC curve; GSEA, Gene set enrichment analysis.
