# Supplementary material for: Developing the novel diagnostic model and potential drugs by integrating bioinformatics and machine learning for aldosterone-producing adenomas
Source: Front Mol Biosci. 2024 Jan 4;10:1308754. doi: 10.3389/fmolb.2023.1308754 (PMC10794617; doi:10.3389/fmolb.2023.1308754)
Supplement: Supplementary file 1 [file Table1.DOCX]

**1.batch de-effect**

library(limma)

library(sva)

library(tinyarray)

files=c(".txt", ".txt")

geneList=list()

for(i in 1:length(files)){

fileName=files[i]

rt=read.table(fileName, header=T, sep="\t", check.names=F)

header=unlist(strsplit(fileName, "\\.|\\-"))

geneList[[header[1]]]=as.vector(rt[,1])

}

intersectGenes=Reduce(intersect, geneList)

allTab=data.frame()

batchType=c()

for(i in 1:length(files)){

fileName=files[i]

header=unlist(strsplit(fileName, "\\.|\\-"))

rt=read.table(fileName, header=T, sep="\t", check.names=F)

rt=as.matrix(rt)

rownames(rt)=rt[,1]

exp=rt[,2:ncol(rt)]

dimnames=list(rownames(exp),colnames(exp))

data=matrix(as.numeric(as.matrix(exp)), nrow=nrow(exp), dimnames=dimnames)

rt=avereps(data)

colnames(rt)=paste0(header[1], "_", colnames(rt))

qx=as.numeric(quantile(rt, c(0, 0.25, 0.5, 0.75, 0.99, 1.0), na.rm=T))

LogC=( (qx[5]>100) || ( (qx[6]-qx[1])>50 && qx[2]>0) )

if(LogC){

rt[rt<0]=0

rt=log2(rt+1)}

rt=normalizeBetweenArrays(rt)

if(i==1){

allTab=rt[intersectGenes,]

}else{

allTab=cbind(allTab, rt[intersectGenes,])

}

}

sample=read.table(".txt", header=F, sep="\t", check.names=F,row.names = 1)

allTab=allTab[,rownames(sample)]

allTabOut=rbind(geneNames=colnames(allTab), allTab)

write.table(allTabOut, file="raw.exp.txt", sep="\t", quote=F, col.names=F)

batchType=as.character(as.data.frame(strsplit(rownames(sample), "_"))[1,])

modeType=as.vector(sample[,1])

mod=model.matrix(~as.factor(modeType))

normalizeTab=ComBat(allTab, batchType,mod, par.prior=TRUE)

normalizeTabout=rbind(geneNames=colnames(normalizeTab), normalizeTab)

write.table(normalizeTabout, file="combat.exp.txt", sep="\t", quote=F, col.names=F)

type=factor(x = colnames(allTab),labels =modeType)

summary(type)

pdf(file = "raw_PCA_Type.pdf",width=5.5,height = 4.5)

draw_pca(allTab,type)

dev.off()

type=factor(x = colnames(allTab),labels =batchType)

summary(type)

pdf(file = "raw_PCA_Group.pdf",width=5.5,height = 4.5)

draw_pca(allTab,type)

dev.off()

type=factor(x = colnames(normalizeTab),labels =modeType)

summary(type)

pdf(file = "nor_PCA_Type.pdf",width=5.5,height = 4.5)

draw_pca(normalizeTab,type)

dev.off()

type=factor(x = colnames(normalizeTab),labels =batchType)

summary(type)

pdf(file = "nor_PCA_Group.pdf",width=5.5,height = 4.5)

draw_pca(normalizeTab,type)

dev.off()

**2.DEG**

library(tidyverse)

library(GEOquery)

library(stringr)

exp <- read.table("exp.txt",sep = "\t",row.names = 1,check.names = F,stringsAsFactors = F,header = T)

pdata <- read.table(".txt",sep = "\t",check.names = F,stringsAsFactors = F,header = T,row.names = 1)

group_list <- ifelse(str_detect(pdata$group, "APA"), "tumor",

"normal")

group_list = factor(group_list,

levels = c("normal","tumor"))

library(limma)

design=model.matrix(~group_list)

fit=lmFit(exp,design)

fit=eBayes(fit)

deg=topTable(fit,coef=2,number = Inf)

write.table(deg, file = "deg_all.txt",sep = "\t",row.names = T,col.names = NA,quote = F)

**3.LASSO**

library(survival)

library(glmnet)

library(ggplot2)

library(ggsci)

library(patchwork)

library(limma)

inputFile=".txt"

C="C"

rt=read.table(inputFile, header=T, sep="\t", check.names=F)

rt=as.matrix(rt)

rownames(rt)=rt[,1]

exp=rt[,2:ncol(rt)]

dimnames=list(rownames(exp),colnames(exp))

data=matrix(as.numeric(as.matrix(exp)),nrow=nrow(exp),dimnames=dimnames)

data=avereps(data)

data=t(data)

data=data[,read.table("disease.txt", header=F, sep="\t", check.names=F)[,1]]

sample=read.table("sample.txt",sep="\t",header=F,check.names=F,row.names = 1)

data=data[rownames(sample),]

x=as.matrix(data)

afcon=sum(sample[,1]==C)

group=c(rep("0",afcon),rep("1",nrow(data)-afcon))

group=as.matrix(group)

rownames(group)=rownames(data)

y=as.matrix(group[,1])

set.seed(123)

cvfit = cv.glmnet(x, y,family = "binomial", nlambda=100, alpha=1,nfolds = 10)

fit <- glmnet(x,y,family = "binomial")

cvfit$lambda.min

coef <- coef(fit, s = cvfit$lambda.min)

index <- which(coef != 0)

actCoef <- coef[index]

lassoGene=row.names(coef)[index]

geneCoef=cbind(Gene=lassoGene, Coef=actCoef)

write.table(geneCoef, file="geneCoef.xls", sep="\t", quote=F, row.names=F)

write.table(file="lassoset.txt",lassoGene,sep="\t",quote=F,col.names=F,row.names=F)

pdf("lasso.pdf",height = 5,width = 7)

layout(matrix(c(1,1,2,2), 2, 2, byrow = F))

plot(fit,xvar = 'lambda')

plot(cvfit)

abline(v=log(c(cvfit$lambda.min,cvfit$lambda.1se)),lty="dashed")

dev.off()

**4.randomForest**

library(randomForest)

library(limma)

library(ggpubr)

set.seed(123)

inputFile=".txt"

C="C"

rt=read.table(inputFile, header=T, sep="\t", check.names=F)

rt=as.matrix(rt)

rownames(rt)=rt[,1]

exp=rt[,2:ncol(rt)]

dimnames=list(rownames(exp),colnames(exp))

data=matrix(as.numeric(as.matrix(exp)),nrow=nrow(exp),dimnames=dimnames)

data=avereps(data)

data=t(data)

data=data[,read.table("disease.txt", header=F, sep="\t", check.names=F)[,1]]

sample=read.table("sample.txt",sep="\t",header=F,check.names=F,row.names = 1)

data=data[rownames(sample),]

colnames(data)=gsub("-", "afaf", colnames(data))

afcon=sum(sample[,1]==C)

group=c(rep("con",afcon),rep("treat",nrow(data)-afcon))

rf=randomForest(as.factor(group)~., data=data, ntree=500)

pdf(file="forest.pdf", width=6, height=6)

plot(rf, main="Random forest", lwd=2)

dev.off()

optionTrees=which.min(rf$err.rate[,1])

optionTrees

rf2=randomForest(as.factor(group)~., data=data, ntree=optionTrees)

importance=importance(x=rf2)

importance=as.data.frame(importance)

importance$size=gsub("-", "afaf", importance$size)

importance$size=rownames(importance)

importance=importance[,c(2,1)]

names(importance)=c("Gene","importance")

af=importance[order(importance$importance,decreasing = T),]

af=af[1:20,]

p=ggdotchart(af, x = "Gene", y = "importance",

color = "importance", # Custom color palette

sorting = "descending",

add = "segments",

add.params = list(color = "lightgray", size = 2),

dot.size = 6,

font.label = list(color = "white", size = 9,

vjust = 0.5),

ggtheme = theme_bw() ,

rotate=TRUE )

p1=p+ geom_hline(yintercept = 0, linetype = 2, color = "lightgray")+

gradient_color(palette =c(ggsci::pal_npg()(2)[2],ggsci::pal_npg()(2)[1]) ) +

grids()

pdf(file="importance.pdf", width=6, height=6)

print(p1)

dev.off()

rfGenes=importance[order(importance[,"importance"], decreasing = TRUE),]

write.table(rfGenes, file="rfGenes.xls", sep="\t", quote=F, col.names=T, row.names=F)

**5.SVM-RFE**

library(tidyverse)

library(glmnet)

source('msvmRFE.R')

library(VennDiagram)

library(sigFeature)

library(e1071)

library(caret)

library(randomForest)

library(limma)

inputFile=".txt"

C="C"

rt=read.table(inputFile, header=T, sep="\t", check.names=F)

rt=as.matrix(rt)

rownames(rt)=rt[,1]

exp=rt[,2:ncol(rt)]

dimnames=list(rownames(exp),colnames(exp))

data=matrix(as.numeric(as.matrix(exp)),nrow=nrow(exp),dimnames=dimnames)

data=avereps(data)

data=t(data)

data=data[,read.table("disease.txt", header=F, sep="\t", check.names=F)[,1]]

sample=read.table("sample.txt",sep="\t",header=F,check.names=F,row.names = 1)

data=data[rownames(sample),]

afcon=sum(sample[,1]==C)

group=c(rep("0",afcon),rep("1",nrow(data)-afcon))

group=as.matrix(as.numeric(group))

rownames(group)=rownames(data)

colnames(group)="Type"

input <- as.data.frame(cbind(group,data))

input$Type=as.factor(input$Type)

svmRFE(input, k = 10, halve.above = 100)

nfold = 10

nrows = nrow(input)

folds = rep(1:nfold, len=nrows)[sample(nrows)]

folds = lapply(1:nfold, function(x) which(folds == x))

results = lapply(folds, svmRFE.wrap, input, k=10, halve.above=100)

top.features = WriteFeatures(results, input, save=F) head(top.features)

write.csv(top.features,"feature_svm.csv")

featsweep = lapply(1:X, FeatSweep.wrap, results, input)

no.info = min(prop.table(table(input[,1])))

errors = sapply(featsweep, function(x) ifelse(is.null(x), NA, x$error))

pdf("svm-error.pdf",width = 5,height = 5)

PlotErrors(errors, no.info=no.info)

dev.off()

pdf("svm-accuracy.pdf",width = 5,height = 5)

Plotaccuracy(1-errors,no.info=no.info)

dev.off()

which.min(errors)

**6.ssGSEA**

library(GSVA)

library(limma)

library(GSEABase)

expFile=".txt"

gmtFile=""

rt=read.table(expFile, header=T, sep="\t", check.names=F)

rt=as.matrix(rt)

rownames(rt)=rt[,1]

exp=rt[,2:ncol(rt)]

dimnames=list(rownames(exp),colnames(exp))

mat=matrix(as.numeric(as.matrix(exp)),nrow=nrow(exp),dimnames=dimnames)

mat=avereps(mat)

mat=normalizeBetweenArrays(mat)

mat=mat[rowMeans(mat)>0,]

geneSet=getGmt(gmtFile, geneIdType=SymbolIdentifier())

ssgseaScore=gsva(mat, geneSet, method='ssgsea', kcdf='Gaussian', abs.ranking=TRUE)

normalize=function(x){

return((x-min(x))/(max(x)-min(x)))}

ssgseaOut=normalize(ssgseaScore)

ssgseaOut=rbind(id=colnames(ssgseaOut),ssgseaOut)

write.table(ssgseaOut, file="ssGSEAscore.txt", sep="\t", quote=F, col.names=F)

**7.GSEA**

library(ggplot2)

library(limma)

library(pheatmap)

library(ggsci)

lapply(c('clusterProfiler','enrichplot','patchwork'), function(x) {library(x, character.only = T)})

library(org.Hs.eg.db)

library(patchwork)

expFile=".txt"

hub="LASSO.txt"

rt=read.table(expFile,sep="\t",header=T,check.names=F)

rt=as.matrix(rt)

rownames(rt)=rt[,1]

exp=rt[,2:ncol(rt)]

dimnames=list(rownames(exp),colnames(exp))

data=matrix(as.numeric(as.matrix(exp)),nrow=nrow(exp),dimnames=dimnames)

data=avereps(data)

geneaf=read.table(hub,sep="\t",header=F,check.names=F)[,1]

for (genei in geneaf) {

group <- ifelse(data[c(genei),]> median(data[c(genei),]), "High", "Low")

group <- factor(group,levels = c("High","Low"))

design <- model.matrix(~0+group)

colnames(design) <- levels(group)

fit <- lmFit(data,design)

cont.matrix<-makeContrasts(High-Low,levels=design)

fit2 <- contrasts.fit(fit, cont.matrix)

fit2 <- eBayes(fit2)

deg=topTable(fit2,adjust='fdr',number=nrow(data))

Diff=deg

Diff=Diff[order(as.numeric(as.vector(Diff$logFC))),]

diffGene=as.vector(rownames(Diff))

diffLength=length(diffGene)

afGene=c()

if(diffLength>(60)){

afGene=diffGene[c(1:30,(diffLength-30+1):diffLength)]

}else{

afGene=diffGene

}

afExp=data[afGene,]

Type1=as.data.frame(group)

Type1=Type1[order(Type1$group,decreasing = T),,drop=F]

Type=Type1[,1]

names(Type)=rownames(Type1)

Type=as.data.frame(Type)

anncolor=list(Type=c(High="red",Low="blue" ))

logFC_t=0

deg$g=ifelse(deg$P.Value>0.05,'stable',

ifelse( deg$logFC > logFC_t,'UP',

ifelse( deg$logFC < -logFC_t,'DOWN','stable') )

)

table(deg$g)

deg$symbol=rownames(deg)

df <- bitr(unique(deg$symbol), fromType = "SYMBOL",

toType = c( "ENTREZID"),

OrgDb = org.Hs.eg.db)

DEG=deg

DEG=merge(DEG,df,by.y='SYMBOL',by.x='symbol')

data_all_sort <- DEG %>%

arrange(desc(logFC))

geneList = data_all_sort$logFC

names(geneList) <- data_all_sort$ENTREZID

head(geneList)

kk2 <- gseKEGG(geneList = geneList,

organism = 'hsa',

nPerm = 10000,

minGSSize = 10,

maxGSSize = 200,

pvalueCutoff = 0.05,

pAdjustMethod = "none" )

class(kk2)

colnames(kk2@result)

kegg_result <- as.data.frame(kk2)

rownames(kk2@result)[head(order(kk2@result$enrichmentScore))]

af=as.data.frame(kk2@result)

write.table(af,file=paste0("2.",paste0(genei,"_all_GSEA.xls")),sep="\t",quote=F,col.names=T)

num=5

pdf(paste0("2.",paste0(genei,"_down_GSEA.pdf")),width = 8,height = 8)

af=gseaplot2(kk2, geneSetID = rownames(kk2@result)[head(order(kk2@result$enrichmentScore),num)])

print(af)

dev.off()

pdf(paste0("2.",paste0(genei,"_up_GSEA.pdf")),width = 8,height = 8)

af=gseaplot2(kk2, geneSetID = rownames(kk2@result)[tail(order(kk2@result$enrichmentScore),num)])

print(af)

dev.off()

num=5

pdf(paste0("2.",paste0(genei,"_all_GSEA.pdf")),width = 4,height = 5)

af=gseaplot2(kk2, geneSetID = rownames(kk2@result)[c(head(order(kk2@result$enrichmentScore),num),tail(order(kk2@result$enrichmentScore),num))])

print(af)

dev.off()

}

**8.ANN**

zscale=read.table()

ind<-sample(2,nrow(zscale),replace = T,prob = c()

trainset<-zscale[ind==1,]

testset<-zscale[ind==2,]

trainset$normal<-trainset$group=="normal"

trainset$tumor<-trainset$group=="tumor"

trainset1 = trainset[,2:6]

testset1 = testset[,2:4]

library(neuralnet)

library(NeuralNetTools)

network<-neuralnet(normal + tumor ~ X + X +X ,trainset1,hidden = c(8,3,2))

par(cex = 0.8)

plotnet(network,pos_col = "red", neg_col = "grey")

par(mfrow=c(2,3))

gwplot(network,selected.covariate = "X")

gwplot(network,selected.covariate = "X")

gwplot(network,selected.covariate = "X")

net.predict1<-compute(network,trainset1[,2:4])$net.result

net.prediction1<-c("normal","tumor")[apply(net.predict1,1,which.max)]

predict.table1<-table(trainset$group,net.prediction1)

net.predict<-compute(network,testset[,2:4])$net.result

net.prediction<-c("normal","tumor")[apply(net.predict,1,which.max)]

predict.table<-table(testset$group,net.prediction)

write.table (trainset,file ="trainset.txt", row.names = T, col.names =T, quote =FALSE)

write.table (testset,file ="testset.txt", row.names = T, col.names =T, quote =FALSE)

cbind(net.prediction1,trainset[,2])

cbind(net.prediction,testset[,2])

predict.table1

net.prediction1

net.predict1

confusionMatrix(predict.table1)

predict.table

net.prediction

net.predict
